# Nurse-targeted care for HIV positive persons with CD4<100 improved time to ART initiation and retention in Uganda

**DOI:** 10.1186/1748-5908-10-S1-A81

**Published:** 2015-08-20

**Authors:** Agnes N Kiragga, Elizabeth Nalintya, Bozena Morawski, Joanita Kigozi, Benjamin J Park, Jonathan E Kaplan, David R Boulware, David B Meya, Yukari C Manabe

**Affiliations:** 1 Infectious Diseases Institute, College of Health Sciences, Makerere University, Kampala, 256, Uganda; 2 Division of Infectious Disease and International Medicine, Department of Medicine, University of Minnesota, Minneapolis, MN, 55455, USA; 3 Division of Global HIV/AIDS (DGHA), Center for Global Health (CGH), Centers for Disease Control and Prevention, Atlanta, GA, 30333, USA; 4 School of Medicine, College of Health Sciences, Makerere University, Kampala, 256, Uganda; 5 Division of Infectious Diseases, Department of Medicine, Johns Hopkins University School of Medicine, Baltimore, MD, 21205, USA

## Background

HIV testing and retention in care treatment are key strategies towards an AIDS free generation. Immediate treament of HIV patients with the lowest CD4 T cell counts is critical given their high rates of pre-antiretroviral therapy mortality. We evaluated the impact of adding an additional nurse-counsellor on HIV outcomes among patients enrolled in care in 7 urban public clinics in Kampala, Uganda.

## Methods

One additional research nurse per clinic was specifically tasked with identifying and tracking HIV-infected persons with CD4<100 cells/μL who were lost, and expediting ART initiation among patients enrolled after July 2012. Data were also retrospectively collected on all patients with CD4<100 cells/μL that registered at these clinics in the latter six months of 2011. We compared time from CD4 blood draw to ART initiation, frequency of CD4 testing and 6 month retention-in-care by time period. Analyses of categorical and continuous variables were conducted using Χ2 and Mann-Whitney tests.

## Results

A total of 258 patients in the 2011 cohort and 593 in the 2012 cohort completed 6 months from clinic registration. Median age of patients in both cohorts was 32 years and 55% were female. Median CD4 cells/μL [Interquartile, IQR] count in the 2011 cohort was 34 [13, 61] and 43 [19, 71] in 2012 (p < 0.002). Median days from CD4 blood draw to ART initiation reduced from 42 [IQR: 28, 56] in the 2011 to 33 [IQR: 21, 47] in 2012, p < 0.001. Six-month retention in care was 62.0% (160/258) in 2011 cohort compared to 75.9% (450/593) in 2012 (p < 0.001). Among the patients retained in care, 24.4% (39/160) received a CD4 count test at 6 months versus 53.3% (240/450) in the 2012 cohort.

## Conclusion

The addition of one nurse per clinic to identify and follow severely immune-compromised new clinic patients with CD4<100 led to improved patient care and better outcomes. Funder: The study is an Implementation Science project funded by CDC.

